# Early miR-320b and miR-25-3p miRNA levels correlate with multiple sclerosis severity at 10 years: a cohort study

**DOI:** 10.1186/s12974-023-02816-8

**Published:** 2023-06-01

**Authors:** Alicia Gonzalez-Martinez, Gauruv Bose, Hrishikesh Lokhande, Shrishti Saxena, Brian C. Healy, Mariann Polgar-Turcsanyi, Howard L. Weiner, Tanuja Chitnis

**Affiliations:** 1 Translational Neuroimmunology Research Center (TNRC), Ann Romney Center for Neurologic Diseases (ARCND), Department of Neurology, Brigham and Women’s Hospital, 60 Fenwood Road, 9002K, Boston, MA 02115 USA; 2 Brigham MS Center, Department of Neurology, Brigham and Women’s Hospital, Boston, MA 02115 USA; 3 Harvard Medical School, Boston, MA 02115 USA

**Keywords:** miRNA, Long-term, Disability, EDSS, miR-320b, miR-25-3p, Multiple sclerosis, Biomarker, RRMS, SPMS

## Abstract

**Background:**

Multiple sclerosis (MS) is a chronic demyelinating autoimmune disorder which may cause long-term disability. MicroRNA (miRNA) are stable, non-coding molecules that have been identified in our Comprehensive Longitudinal Investigation of Multiple Sclerosis at the Brigham and Women’s Hospital (CLIMB)-cohort, as well as other international cohorts, as potential disease biomarkers in MS. However, few studies have evaluated the association of miRNA expression early in the MS disease course with long-term outcomes. Therefore, we aimed to evaluate the potential role of three candidate serum miRNAs previously correlated with MS disability in patients with MS, miR-320b, miR-25-3p and miRNA 486-5p, as early biomarkers of MS disability at 10-year follow-up.

**Main body:**

We included 144 patients with serum obtained within three years of MS onset. miRNA expression was measured by RNA extraction followed by RT-PCR. Demographic, clinical, brain MRI and other biomarkers were collected. The primary outcome was the association between early miRNA expression and retaining benign MS, defined as EDSS ≤ 2 at 10-year follow-up. Among the 144 patients, 104 were benign and 40 were not benign at 10-year follow-up. 89 (62%) were women, with mean age at onset 37.7 (SD: 9.6) years. Patients who retained benign MS had lower values of miR-25-3p (*p* = 0.047) and higher miR-320b (*p* = 0.025) values. Development of SPMS was associated with higher miR-320b (*p* = 0.002) levels. Brain parenchymal fraction at year 10 was negatively correlated with miR-25-3p (*p* = 0.0004) and positively correlated with miR-320b (*p* = 0.006). No association was found between miR-486-5p and any outcome, and 10-year T2-lesion volume was not associated with any miRNA.

**Conclusions:**

Our results show that miR-320b and miR-25-3p expression are early biomarkers associated with MS severity and brain atrophy. This study provides class III evidence of that miR-320b and miR-25-3p are associated with long-term MS disability which may be a potential tool to risk-stratify patients with MS for early treatment decisions.

**Supplementary Information:**

The online version contains supplementary material available at 10.1186/s12974-023-02816-8.

## Background

Multiple sclerosis (MS) is an immune-mediated disease of the central nervous system (CNS) characterized by inflammation, demyelination, and neurodegeneration [1].

Identifying patients at greater risk for a worse disease course is a challenge and may have implication on treatment selection. Although important efforts to discover new biomarkers of worse disease prognosis have been made in the past decades, further study is needed to evaluate how biomarkers measured early after MS onset may help clinicians predict long-term outcomes [2]. Moreover, EDSS of 2.0 at year 10 lead to more favorable long-term outcomes, while patients with an EDSS of 2.5 or higher were more likely to continue to demonstrate disability worsening [3].

MicroRNAs (miRNAs) are small non-coding single-stranded RNA molecules with the ability to regulate gene expression at the post-transcriptional level by binding to specific messenger RNA [4]. Furthermore, miRNAs are stable after repeated freeze–thaw cycles and long-term storage, which makes them good candidates for research and clinical use [5]. Importantly, several miRNAs have been proposed as potential biomarkers in MS, but the association between the level expressed early after disease onset and long-term outcomes has not been explored [5].

Previous studies performed in our Comprehensive Longitudinal Investigation of Multiple Sclerosis at Brigham and Women’s Hospital (CLIMB) cohort have found a set of miRNA associated with MS [6–8]. Validation studies in external international cohorts as part of the Serially Unified Multicenter Multiple Sclerosis Investigation (SUMMIT) collaboration effort confirmed that three miRNAs (miR-320b, miR-25-3p, and miR-486-5p) are differentially expressed between MS patients and healthy controls as well as between MS patients and other neurological conditions including asthma, rheumatoid arthritis, amyotrophic lateral sclerosis (ALS) and Alzheimer’s disease (AD), and are associated with the Expanded Disability Status Scale (EDSS) [6, 8] and correlated with disease severity on MRI [9].

The objective of this study was to evaluate the association of miR-25-3p, 320b and 486-5p with MS severity by clinical and MRI measurements at 10-year follow-up.

## Methods

### Study design and participants

This study was a retrospective analysis of prospectively collected data from patients enrolled in CLIMB, an observational, longitudinal cohort study, that has been following more than 2000 patients diagnosed with MS with biannual clinical examinations, annual MRI scans, and serum samples obtained on a yearly basis. Patients were included in this study if they had a diagnosis of relapsing–remitting MS by 2017 McDonald Criteria, their first visit and blood sample donation within 3 years of symptom onset, and 10-year follow-up evaluation.

### Variables included in the study

We collected demographical variables such as sex, age, and clinical variables such as EDSS at baseline. Our primary outcome was retaining benign MS, defined as having retained an EDSS ≤ 2 at 10-year follow-up, based on an analysis that suggest favorable outcomes beyond this timeframe in patients with this EDSS [3], otherwise, patients were defined as not benign. Secondary outcomes included development of secondary progressive (SP)MS, and 10-year MRI parameters brain parenchymal fraction (BPF) and T2-lesion volume (T2LV).

### Serum samples and methods

Blood samples were collected in glass red-top serum vacutainer tubes without additives (BD, Franklin Lakes, NJ); and kept at room temperature for 30–60 min before centrifugation at 2000 rpm for 10 min to separate serum, and then stored at – 70 °C until RNA extraction. Serum was frozen within 2 h of the blood draw.

### RNA isolation, RT, and qPCR

RNA was isolated using the miRcury Qiagen kit according to the manufacturer’s instructions from initial volume of 220 mcL. RNA was converted to complementary DNA using a synthesis kit from Qiagen following the manufacturer’s instructions. Prepared complementary DNAs were stored at -20 °C until use. We used miRCURY LNA miRNA PCR Assays (Additional file 1: Table S3). A positive signal was defined by a threshold cycle (Ct) cut-off value of < 36 cycles in the PCR run. All samples were measured in technical duplicates and mean values were obtained. Quality control measures for each step according to serum miRNA studies recommendations were applied during analysis [10] and are included in Additional file 1: Table S2. Data were normalized using the geomean of all miRNA and miRNA relative quantity (RQ) are indicated [11].

### Brain MRI measurements

At 10-year follow-up, patients underwent brain MRI on a 3 T Siemens Skyra scanner. We consistently obtained three 3D high-resolution sequences with T1-, T2- and fluid-attenuated inversion-recovery (FLAIR)-weighting at sagittal 1 mm voxels, as well as T1-weighted gradient echo (TR/TE/TI: 2300/2.96/900 ms, flip angle: 9°), T2-spin echo (TR/TE: 2500/300 ms) and T2-FLAIR (TR/TE/TI: 5000/389/1800 ms) [12]. Brain T2LV and BPF were processed from the 3-channel high-resolution sequences, using a previously described in-house pipeline [13].

### Other biomarker measurements

Additionally, we measured cytokines (IL1b, IL2, IL-4, IL-5, IL-8, IL10, IL-17a, IL-18), IFN-gamma, TNF-alpha, TGF-beta, neurofilament light chain (NfL), and glial fibrillary acidic protein (GFAP), and chitinase 3 like 1 (CHI3L1). All samples were measured in technical duplicates and mean values were obtained. We used an ultrasensitive single-molecule array assay (Simoa instrument (Quanterix); UmanDiagnostics) to measure serum NfL and GFAP levels, as previously described [14]. We used the automated ELISA platform, ELLA, high sensitivity immunoassay system by ProteinSimple to measure cytokine and growth factor levels.

### miRNA target

We performed in silico miRNA target prediction analysis using DIANA-miRPath v3.0 [15]. Among the numerous pathways involved, to identify pathways that might be regulated by the miRNAs that were differentially expressed between the groups compared, we used the Kyoto Encyclopedia of Genes Genomes (KEGG) pathway analysis. The pathway intersection analysis method combines the results of pathway enrichment analyses of multiple gene sets to identify the common biological pathways or processes that are perturbed in a given condition, in this case, MS. This approach is useful for identifying key biological pathways that can provide insights into the underlying mechanisms of disease development and progression. Moreover, pathway analysis can also help to identify potential therapeutic targets and biomarkers for disease diagnosis and prognosis [1].

### Statistical analysis

We compared miRNA levels between benign versus not benign groups at 10-year follow-up using a two-sided nonparametric test Wilcoxon rank sum test. Proportional odds models were adjusted for age, sex, and smoking status as “ever smoker” or not. All miRNAs were rank ordered based on the *p*-value. To explore the association between the RQ levels of miRNA and the MRI parameters, BPF and T2LV at 10-year follow-up, Spearman correlation analysis were performed. *p*-values less than 0.05 were considered significant. Receiver operating characteristic curves were generated for specificity and sensitivity values and areas under the curves (AUCs) were calculated (GraphPad Prism). Statistical analysis was completed using the statistical packages R (www.r-project.org) and Stata/IC version 17 (www.stata.com).

### Data availability

Anonymized data not published within this article will be made available by request from any qualified investigator.

### Standard protocol approvals and consent forms

A signed informed consent was received from all the patients. Secondary use approval was obtained from the institutional review board of the Brigham and Women’s Hospital for the CLIMB cohort study. This study has been approved by our hospital’s Ethics Committee and follows the STARD guidelines.

## Results

### Patient characteristics

A total of 144 patients were included. Among them 104 were categorized as benign and 40 as not benign MS at 10-year follow-up. Mean age at MS onset was 37.3 years (SD: 9.69), 92 (63.8%) were female, and the mean EDSS at baseline was 1.45 (SD: 1.48), with 119 patients (82.6%) with first EDSS 2.0 or less. The demographic characteristics of the groups, long-term clinical and MRI outcomes are shown in Table 1 and Additional file 1: Table S1.

**Table 1:** Patient characteristics

Variable	Total population (N = 144)
Baseline	
Age, years	37.7 (± 9.6)
Female (%)	89 (62%)
Ever smoker (%)	66 (65%)
EDSS at baseline	1.45 (± 1.48)
Duration from MS onset to miRNA sample, years	1.17 (± 0.65)
Untreated at miRNA sample	25 (17.4%)
T2LV at baseline (cm^3^, *N* = 121)	4.09 (± 3.06)
BPF at baseline (*N* = 121)	0.88 (± 0.04)
10-year follow-up	
EDSS	1.88 (± 1.64)
Benign	104 (72.2%)
Not benign	40 (27.8%)
T2LV (cm^3^, *N* = 137)	5.45 (± 6.02)
BPF (*N* = 137)	0.84 (± 0.04)
Untreated at 10-year follow-up	3 (2%)

### Association between benign MS and microRNA levels

**Fig. 1:**
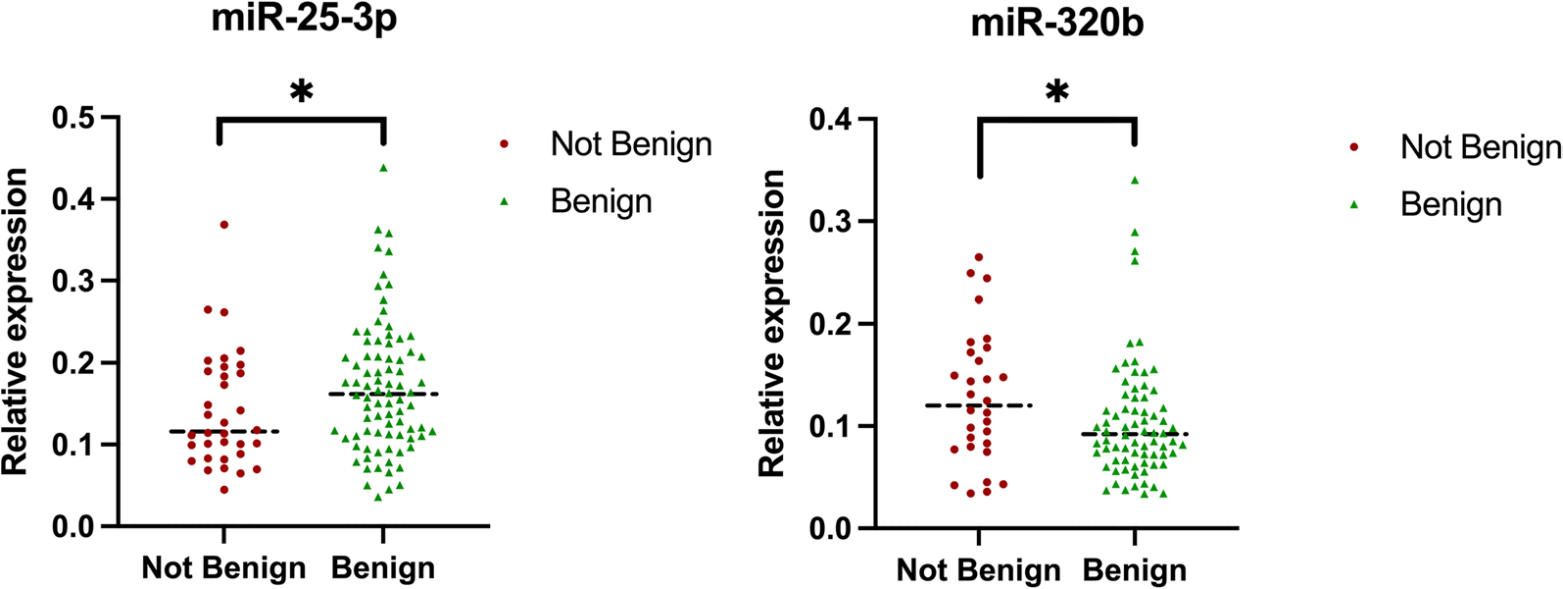
Differential expression of MS deregulated serum miRNA in patients with benign MS vs patients with not benign MS after 10 years follow-up. The MS cohort includes benign and not benign MS at 10 years. Data correspond to the relative levels of indicated miRNA with respect to the geomean of all miRNA. The y-axis depicts RQ values normalized to the geomean of all miRNA; black bars indicate median values; *p* < 0.05, **miRNAs = microRNAs; MS = multiple sclerosis. RQ: relative quantity

**Fig. 2:**
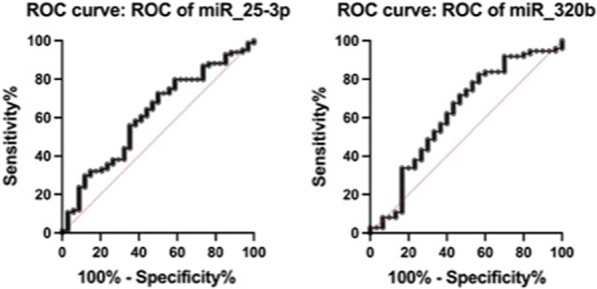
Receiving operating curves of baseline miRNAs differentially expressed between benign and not benign MS at year 10. AUC miR 25-3p: 0.61; AUC miR 320b: 0.62

Higher RQ levels of miR-25-3p at baseline were found in patients with benign MS at 10 years while lower RQ levels of miR-320b at baseline were found in patients with benign MS at 10 years after adjustment of age, sex, smoking status and treatment at baseline (Table 2 and Fig. 1). There was no impact of treatment status in miRNAs expression levels. Receiver operating characteristic curves are included (Fig. 2).

**Table 2:** Multivariate regression model adjusted by age, sex, smoke and treatment at baseline regarding benign or non-benign MS

miRNA	Not benign	Benign	Adjusted *p* value
25-3p	0.11	0.16	0.047*
320b	0.12	0.09	0.025*
486-5p	1.93	2.13	0.426

Median of relative levels of miRNA expression normalized to the geomean. *p < 0.05. Adjusted *p* values for sex, age, smoke and treatment at baseline

In addition, an association between miR-320b and SPMS at year 10 (*p* = 0.0272) in the multivariate model after adjustment of age, sex, and smoking status. No association was found between miR-25-3p (*p* = 0.454) and miR-486-3p (*p* = 0.981) and SPMS at year 10.

### Correlation between MRI outcomes and miRNA levels

Levels of miR-25-3p negatively correlated with BPF at year 10 (Spearman correlation coefficient r_s_ = − 0.326; *p* = 0.0004) while levels of miR-320b positively correlated with BPF at year 10 (Spearman correlation coefficient r_*s*_ = 0.270; *p* = 0.006). There was no correlation between miR-486-5p and BPF (Spearman correlation coefficient r_s_ = − 0.055; *p* = 0.535).

Regarding miRNA and T2LV at year 10, no correlations were found between miR-25-3p (Spearman correlation coefficient r_s_ = − 0.01; *p* = 0.872), miR-320b (Spearman correlation coefficient r_s_ = − 0.02; *p* = 0.815) or miR-486-5p (Spearman correlation coefficient r_s_ = 0.166; *p* = 0.062).

### Correlation miRNA and other serum biomarkers

Levels of miR-25-3p negatively correlated with IL-17a, IL-1b and IL-18 (Table 3). Levels of miR-486-5p negatively correlated with TNFa and IL-18 and positive correlated with IL-10. Levels of miR-320b did not correlate with any cytokine. We did not find an association between NfL, GFAP or CHI3L1 and miRNA levels. The main results are summarized in Tables 3 and 4. All the correlations between miRNA and the other biomarkers are included in Additional file 1: Tables S4–S6).

**Table 3:** Cytokines associated with MS-regulated miRNA

	hsa.miR.25.3p	hsa.miR.320b	hsa.miR.486.5p
TNFa, *p* value (Rho)	0.645 (0.043)	0.267 (− 0.111)	0.003** (− 0.265)
Il-17a, *p* value (Rho)	0.043* (− 0.192)	0.8275 (0.022)	0.050 (**− **0.175)
Il-10, *p* value (Rho)	0.452 (− 0.071)	0.811 (0.024)	0.036*(0.036)
Il-1b, *p* value (Rho)	0.046* (− 0.189)	0.561 (0.058)	0.395 (− 0.07)
Il-18, *p* value (Rho)	0.028* (− 0.207)	0.980 (0.002)	0.039* (− 0.184)

*TNFa* tumor-necrosis-factor alpha; *p < 0.05; **p < 0.01

**Table 4:**
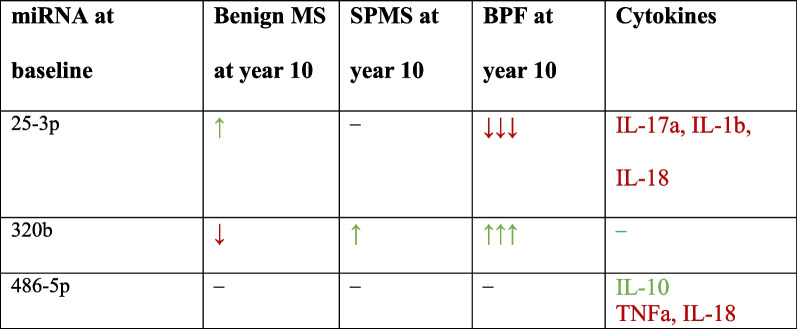
Summary of the relationship between miRNA, MS severity, brain MRI and other serum biomarkers

Summary of the statistically significant associations. SPMS: secondary progressive MS; BPF: brain parenchymal fraction. Arrows represent high (↑) or low (↓) miRNA RQ levels: ↑: *p* < 0.05; ↑↑↑: *p* < 0.001; ↓: *p* < 0.05; ↓↓↓: *p* < 0.001. Red: negative association; green: positive association. –: no association. Neurofilaments, GFAP or chi3l1 were not associated with miRNA

### Target genes and pathways

We found that hippo signaling pathway and adherens junctions pathway are targeted by miR-320b and miR-25-3p performing a pathway intersection analysis with *p*-value threshold: 0.05. There were 18 genes targeted by miR-320b and mIR-25-3p in the adherens junction pathway and 27 genes in the hippo signaling pathway (*p* < 0.05) (Additional file 1: Table S7).

## Discussion

Identifying patients with MS early in their disease course who are at higher risk for a worse outcome is an area of need. miRNAs are non-coding molecules that may have implications in MS pathogenesis and are stable with respect to measurements making them potential candidate biomarkers. In this study, we demonstrated that miR-25-3p and miR-320b measured within 3 years of MS onset are modestly associated with MS severity at 10-year follow-up. This is, to our knowledge, one of the first studies evaluating the role of miRNA as early biomarkers of long-term outcomes in patients with MS.

A strength of this study is that included patients have been well characterized regarding disease course as part of the CLIMB cohort. Since serum samples were donated and frozen early after onset, and long-term follow-up was captured, miRNA-based differentiation based on clinical outcomes was possible.

In our study, higher expression of miR-25-3p was found at baseline in patients with benign MS at 10 years follow-up. Previous studies have pointed out that miR-25-3p is an important biomarker in MS [7]. Studies performed in animal models suggest that miR-25-3p is likely induced by inflammatory mediators during autoimmune neuroinflammation [16]. However, overexpressing miR-25-3p attenuates inflammation in mouse models of atherosclerosis [17]. Moreover, a higher expression of miR-25-3p has been detected in T regulatory cells in patients with MS compared to controls [18]. The different results could be explained by the different sources (animal vs human samples) and different groups of comparison (MS vs control). Indeed, in the present focusing only on MS patients a higher expression of miR-25-3p in serum at baseline is related with benign MS at year 10, which supports the role of miRNAs as early biomarker of long-term outcomes.

We also found that lower levels of miR-320b early after MS in patients with benign MS at 10 years. miR-320b has been previously found to be different between patients and healthy controls. Low expression miR-320b levels have been found in patients with other autoimmune conditions such as psoriasis [19]. Moreover, it has been previously associated with EDSS in MS in our own CLIMB cohort [8]. In this study, we also found a positive association between miR-320b levels and SPMS. Therefore, our results support the potential role of miR-320b as an early biomarker of disease progression.

Moreover, we found that ACTB, TGFBR1, SMAD2, SMAD3, ACTG1, CDH1 and CTNNB1 are common target genes involved in adherens junction and hippo signaling pathway through KEEG analysis. Adherens junction is one of the top five MS-associated pathways from merged proteomic and genomic data analysis [15]. Adherens junction protein CDH1 and TGFBR1 are involved in blood–brain barrier (BBB) dysfunction in MS [20, 21]. Disruption of adherens junctions has been associated with increased BBB permeability, allowing immune cells to enter the brain and spinal cord, contributing to inflammation and tissue damage [22]. Understanding the interactions between these proteins and other cellular pathways may be important for developing new therapies for MS.

On the other hand, hippo signaling pathway is a key regulatory pathway that plays a role in regulating cell proliferation, differentiation, and survival. It is involved in the development and maintenance of tissues and organs, and its dysregulation has been linked to a variety of diseases, including cancer, developmental disorders, and neurological disorders [23].

The association between cigarette smoking and MS has been previously found in our group suggesting that the inflammatory and neurotoxic outcomes may influence the prognosis [24]. Therefore, we adjusted not only by age and sex, but also by smoking.

Regarding brain atrophy, we found a negative correlation between miR-25-3p RQ baseline levels and BPF at year 10, suggesting that higher levels of this miRNA at baseline are associated with lower brain values, at year 10, which could be related with spinal cord lesions leading to a worse prognosis [1]. Moreover, we found a positive correlation between baseline levels of miR-320b and BPF at year 10. These observations suggest a decoupling between miRNA expression and either clinical status or brain atrophy, which might be related with the observed dissociation between focal inflammatory demyelination in white matter and diffuse cerebral neurodegeneration [25]. It may also indicate that lesion location, such as in the spinal cord, may portend to greater clinical disability early in the disease course, as opposed to brain atrophy which may impact cognition and patient function further than 10 years from onset.

Regarding global lesion volume, T2LV did not show an association with miRNA expression levels. In a previous study performed in patients with MS, T2LV showed the weakest association with miRNA expression. This observation is consistent with the notion that T2 lesions are nonspecific for the nature and severity of tissue injury [9].

It has been reported that pro-inflammatory circulating cytokines are increased in patients with MS and may influence the disease activity, while anti-inflammatory circulating cytokines such as IL-4 and IL-10 are reduced and may exert a protective effect [26]. We found that baseline levels of miR-25-3p negatively correlated with pro-inflammatory cytokines suggesting that higher miR-25-3p baseline levels, which are associated with benign MS at year 10, occur in the absence of inflammatory cytokines at baseline. We also found that levels of miR-486-5p negatively correlated with pro-inflammatory cytokines TNFa and IL-18. In addition, miR-486-5p also showed a positive correlation with anti-inflammatory IL-10 suggesting a possible anti-inflammatory regulating role of these two MS-associated miRNA. These results suggest that higher MS-associated miRNA levels do not correlate with a high inflammatory baseline. Moreover, the levels of miR-320b did not correlate with any cytokine level, and none of the MS-associated miRNA show correlation with sNfL, GFAP or CHI3L1, suggesting that miRNA levels may be independent of other biomarker levels such as sNfL, GFAP or chi3l1 and therefore might represent other biological processes underlying MS.

Reports from various disciplines, including oncology, cardiovascular research, and infectious diseases, have demonstrated the potential diagnostic and prognostic value of circulating miRNAs as biomarkers of different diseases [6, 8, 9]. However, the functional role of disease-associated miRNAs remains to be solved. It is unclear whether specific miRNAs are causative, or reflect a response, to certain pathophysiologic processes as distant messengers. Hippo signaling pathway is one of the main pathways in which target genes of both miRNA 320b and 25-3p are involved. Additional functional studies based on these results will help elucidate the functional role of these early novel biomarkers of long-term outcomes.

The main limitations of the current study are the relatively small sample size and limitation on the effect of treatment during each patient’s follow-up period. Although all patients had disease onset over 10 years ago, approval of novel, higher efficacy treatments became available more recently and may have an effect on disease course, independent of baseline miRNA expression. A prospective study with longitudinal evaluation is needed to evaluate how miRNA expression changes with specific MS treatment, and a validation cohort is needed to reproduce our findings. Nevertheless, the results of our study show the potential role of miRNA as early biomarkers of long-term outcomes in MS.

## Supplementary Information

**Additional file 1.** **Table S1**. Treatment breakdown regarding disease outcome. **Table S2**. Quality control criteria for exclusion of miRNA during analysis. **Table S3**. Kits and primer sources/sequences used to detect specific miRNAs. **Table S4**. Correlation between cytokines, sNFL, GFAP and chi3l1 and MS-associated miRNA. **Table S5**. Correlation between cytokines, sNFL, GFAP and chi3l1 and MS-outcome. **Table S6**. Correlation between BPF and MS-outcome. **Table S7**. Genes targeted by miR-320b and mIR-25-3p in the adherens junction pathway and hippo signaling pathway.

## Data Availability

The datasets generated and/or analyzed in the current study are available from the corresponding author on reasonable request from any qualified investigator.

## Abbreviations

AUC: Area under the curve
CI: Confidence interval
miRNAs: MicroRNAs
MS: Multiple sclerosis
SPMS: Secondary progressive multiple sclerosis

## References

[1] Natural history of multiple sclerosis: a unifying concept | Brain | Oxford Academic. Brain 2006;129(Pt 3):606-16. 10.1093/brain/awl007. 10.1093/brain/awl007 16415308

[2] Housley WJ, Pitt D, Hafler DA (2015). Biomarkers in multiple sclerosis. Clin Immunol Orlando Fla.

[3] Sartori A, Abdoli M, Freedman MS (2017). Can we predict benign multiple sclerosis? Results of a 20-year long-term follow-up study. J Neurol.

[4] Mendell JT, Olson EN (2012). MicroRNAs in stress signaling and human disease. Cell.

[5] Pietrasik S, Dziedzic A, Miller E, Starosta M, Saluk-Bijak J (2021). Circulating miRNAs as potential biomarkers distinguishing relapsing-remitting from secondary progressive multiple sclerosis. A review. Int J Mol Sci.

[6] Regev K, Healy BC, Paul A, Diaz-Cruz C, Mazzola MA, Raheja R (2018). Identification of MS-specific serum miRNAs in an international multicenter study. Neurol Neuroimmunol Neuroinflammation..

[7] Bove R, Chitnis T, Cree BA, Tintoré M, Naegelin Y, Uitdehaag BM (2018). SUMMIT (Serially Unified Multicenter Multiple Sclerosis Investigation): creating a repository of deeply phenotyped contemporary multiple sclerosis cohorts. Mult Scler Houndmills Basingstoke Engl..

[8] Regev K, Paul A, Healy B, von Glenn F, Diaz-Cruz C, Gholipour T (2016). Comprehensive evaluation of serum microRNAs as biomarkers in multiple sclerosis. Neurol Neuroimmunol Neuroinflammation.

[9] Regev K, Healy BC, Khalid F, Paul A, Chu R, Tauhid S (2017). Association between serum MicroRNAs and magnetic resonance imaging measures of multiple sclerosis severity. JAMA Neurol.

[10] Galván-Román JM, Lancho-Sánchez Á, Luquero-Bueno S, Vega-Piris L, Curbelo J, Manzaneque-Pradales M (2020). Usefulness of circulating microRNAs miR-146a and miR-16-5p as prognostic biomarkers in community-acquired pneumonia. PLoS ONE.

[11] Chicharro P, Rodríguez-Jiménez P, Llamas-Velasco M, Montes N, Sanz-García A, Cibrian D (2020). Expression of miR-135b in psoriatic skin and its association with disease improvement. Cells.

[12] Valcarcel AM, Linn KA, Khalid F, Vandekar SN, Tauhid S, Satterthwaite TD (2018). A dual modeling approach to automatic segmentation of cerebral T2 hyperintensities and T1 black holes in multiple sclerosis. NeuroImage Clin.

[13] Meier DS, Guttmann CRG, Tummala S, Moscufo N, Cavallari M, Tauhid S (2018). Dual-sensitivity multiple sclerosis lesion and CSF segmentation for multichannel 3T brain MRI. J Neuroimaging.

[14] Disanto G, Barro C, Benkert P, Naegelin Y, Schädelin S, Giardiello A (2017). Serum Neurofilament light: a biomarker of neuronal damage in multiple sclerosis. Ann Neurol.

[15] Vlachos IS, Zagganas K, Paraskevopoulou MD, Georgakilas G, Karagkouni D, Vergoulis T (2015). DIANA-miRPath v3.0: deciphering microRNA function with experimental support. Nucleic Acids Res.

[16] Zare-Chahoki A, Ahmadi-Zeidabadi M, Azadarmaki S, Ghorbani S, Noorbakhsh F (2021). Inflammation in an animal model of multiple sclerosis leads to MicroRNA-25-3p dysregulation associated with inhibition of Pten and Klf4. Iran J Allergy Asthma Immunol.

[17] Yao Y, Sun W, Sun Q, Jing B, Liu S, Liu X (2019). Platelet-derived exosomal MicroRNA-25-3p inhibits coronary vascular endothelial cell inflammation through Adam10 via the NF-κB signaling pathway in ApoE-/- mice. Front Immunol.

[18] De Santis G, Ferracin M, Biondani A, Caniatti L, Rosaria Tola M, Castellazzi M (2010). Altered miRNA expression in T regulatory cells in course of multiple sclerosis. J Neuroimmunol.

[19] Wang Y, Yu X, Wang L, Ma W, Sun Q (2018). miR-320b is down-regulated in psoriasis and modulates keratinocyte proliferation by targeting AKT3. Inflammation.

[20] Atis M, Akcan U, Altunsu D, Ayvaz E, Uğur Yılmaz C, Sarıkaya D, Temizyürek A, Ahıshalı B, Girouard H, Kaya M (2022). Targeting the blood-brain barrier disruption in hypertension by ALK5/TGF-Β type I receptor inhibitor SB-431542 and dynamin inhibitor dynasore. Brain Res.

[21] Katiyar A, Sharma S, Singh TP, Kaur P (2018). Identification of shared molecular signatures indicate the susceptibility of endometriosis to multiple sclerosis. Front Genet.

[22] Argaw AT, Asp L, Zhang J, Navrazhina K, Pham T, Mariani JN, Mahase S, Dutta DJ, Seto J, Kramer EG, Ferrara N, Sofroniew MV, John GR (2012). Astrocyte-derived VEGF-A drives blood-brain barrier disruption in CNS inflammatory disease. J Clin Invest.

[23] Pfleger CM (2017). The hippo pathway: a master regulatory network important in development and dysregulated in disease. Curr Top Dev Biol.

[24] Rosso M, Chitnis T (2020). Association between cigarette smoking and multiple sclerosis: a review. JAMA Neurol.

[25] Tauhid S, Neema M, Healy BC, Weiner HL, Bakshi R (2014). MRI phenotypes based on cerebral lesions and atrophy in patients with multiple sclerosis. J Neurol Sci.

[26] Wang K, Song F, Fernandez-Escobar A, Luo G, Wang JH, Sun Y (2018). The properties of cytokines in multiple sclerosis: pros and cons. Am J Med Sci.

