# Supplementary material for: Early miR-320b and miR-25-3p miRNA levels correlate with multiple sclerosis severity at 10 years: a cohort study
Source: J Neuroinflammation. 2023 Jun 1;20:136. doi: 10.1186/s12974-023-02816-8 (PMC10233999; doi:10.1186/s12974-023-02816-8)
Supplement: Supplementary file 1 — Additional file 1. Table S1. Treatment breakdown regarding disease outcome. Table S2. Quality control criteria for exclusion of miRNA during analysis. Table S3. Kits and primer sources/sequences used to detect specific miRNAs. Table S4. Correlation between cytokines, sNFL, GFAP and chi3l1 and MS-associated miRNA. Table S5. Correlation between cytokines, sNFL, GFAP and chi3l1 and MS-outcome. Table S6. Correlation between BPF and MS-outcome. Table S7. Genes targeted by miR-320b and mIR-25-3p in the adherens junction pathway and hippo signaling pathway. [file 12974_2023_2816_MOESM1_ESM.docx]

**Additional file 1: Table S1. Treatment breakdown regarding disease outcome.**

| medication_status | Agressive | Benign |
| --- | --- | --- |
| Avonex | 2 | 1 |
| Betaseron | 1 | 2 |
| Cellcept | 0 | 1 |
| Copaxone | 8 | 18 |
| Cytoxan | 0 | 1 |
| Gilenya | 2 | 3 |
| Rebif | 4 | 8 |
| Tecfidera (bg-12) | 0 | 2 |
| Tysabri | 1 | 0 |
| Untreated | 22 | 68 |
| Total | 40 | 104 |

**Additional file 1: Table S2. Quality control criteria for exclusion of miRNA during analysis.**

| **Quality process** | **Criteria** | **Number of samples excluded** |
| --- | --- | --- |
| Hemolysis | miR-451-miR-23a >7 Cts | 6 |
| RNA extraction: differences between samples | UniSp2 < 2.5 Cts or >2.5 Cts according to the median | 43 |
| RT to cDNA: differences between samples | UniSp6 < 2.5 Cts o >2.5 Cts according to the median | 0 |

**Additional file 1: Table S3. Kits and primer sources/sequences used to detect specific miRNAs.**

| **Description** | **Reference/GeneGlobeID** | **Catalog No** |
| --- | --- | --- |
| miRNeasy Serum/Plasma Advanced Kit (50 (217204) | Qiagen | 217204 |
| RNA Spike-in kit, UniRT (339390) | Qiagen | 339390 |
| miRCURY LNA RT Kit (339340) | Qiagen | 339340 |
| miRCURY LNA SYBR Green PCR Kit (4000) (339347) | Qiagen | 339347 |
| Unisp2 miRCURY LNA miRNA PCR Assay | YP00203950 | 339306 |
| Unisp6 miRCURY LNA miRNA PCR Assay | YP00203954 | 339306 |
| miR-451a | YP02119305 | 339306 |
| miR23a-3p | YP00204772 | 339306 |
| hsa-miR-25-3p | YP00204361 | 339306 |
| hsa-miR-486-5p | YP00204001 | 339306 |
| hsa-miR-320b | YP02119299 | 339306 |

**Additional file 1: Table S4. Correlation between cytokines, sNFL, GFAP and chi3l1 and MS-associated miRNA.**

|  | **hsa.miR.25.3p** | | **hsa.miR.320b** | | **hsa.miR.486-5p** | |
| --- | --- | --- | --- | --- | --- | --- |
| **Variable** | **(rho value)** | **p-value** | **(rho value)** | **p-value** | **(rho value)** | **p-value** |
| IL2 | -0.1046255 | 0.2722713 | 0.1650543 | 0.1007833 | -0.0836091 | 0.3539205 |
| IL4 | -0.0411473 | 0.6666433 | 0.1045234 | 0.3007013 | -0.0015468 | 0.9863412 |
| IL5 | -0.0661866 | 0.4880863 | -0.1095576 | 0.2778900 | -0.1194292 | 0.1846418 |
| IL8 | 0.0351741 | 0.7127347 | -0.0056230 | 0.9557212 | 0.1752411 | 0.0506154 |
| IL10 | -0.0716861 | 0.4525863 | 0.0241487 | 0.8115047 | -0.1876552 | 0.0361154 |
| IL-11b | -0.1890598 | 0.0458885 | 0.0587674 | 0.5613835 | -0.0766560 | 0.3955024 |
| IL-17a | -0.1917134 | 0.0428707 | 0.0220585 | 0.8275551 | -0.1753809 | 0.0504287 |
| IL-18 | -0.2078243 | 0.0278923 | 0.0024666 | 0.9805689 | -0.1844121 | 0.0395164 |
| TNFa | 0.0439844 | 0.6451660 | -0.1119862 | 0.2673110 | -0.2650826 | 0.0028120 |
| TGFb | -0.0425457 | 0.6560218 | -0.1637002 | 0.1036481 | 0.0401428 | 0.6566952 |
| IFN-g | -0.1440207 | 0.1297767 | 0.1156393 | 0.2519182 | -0.1166401 | 0.1951819 |
| chi3l1 | -0.1363652 | 0.1516665 | 0.0870807 | 0.3889644 | -0.0362335 | 0.6882989 |
| GFAP | -0.0462995 | 0.6278533 | 0.0164544 | 0.8709231 | 0.0492026 | 0.5858186 |
| sNFL | -0.0112072 | 0.9066393 | -0.0325353 | 0.7479473 | 0.0910661 | 0.3124851 |
| IL: Interleukin. TNF: Tumor necrosis factor; chi2l1: chitinase 3 like 1; GFAP: glial fibrillary acidic protein; sNFL: serum neurofilament; | | | | | | |


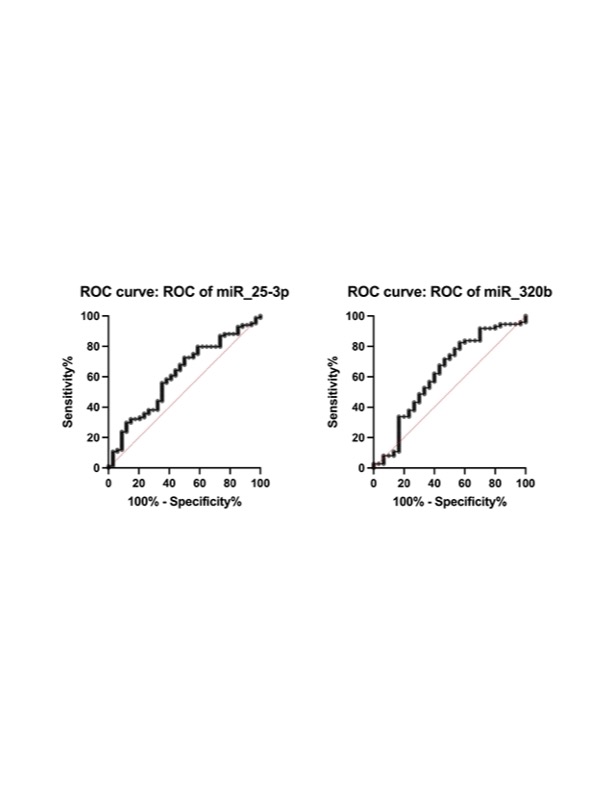


**Additional file 1: Table S5. Correlation between cytokines, sNFL, GFAP and chi3l1 and MS-outcome.**

| marker | estimate | std.error | statistic | p.value |
| --- | --- | --- | --- | --- |
| chi3l1 | -0.000023 | 0.0000106 | -2.17 | 0.0298 |
| gfap | -0.000693 | 0.00348 | -0.199 | 0.842 |
| ifng | -0.0828 | 0.104 | -0.796 | 0.426 |
| il10 | 0.0284 | 0.0393 | 0.723 | 0.47 |
| il17a | -0.0687 | 0.0722 | -0.952 | 0.341 |
| il18 | 0.000198 | 0.00174 | 0.114 | 0.909 |
| il1b | 0.0812 | 0.169 | 0.481 | 0.63 |
| il2 | -0.517 | 0.789 | -0.656 | 0.512 |
| il4 | -1.34 | 0.911 | -1.47 | 0.143 |
| il5 | 0.0175 | 0.0392 | 0.446 | 0.655 |
| il8 | 0.000229 | 0.0006 | 0.382 | 0.702 |
| nfl | 0.0298 | 0.0223 | 1.34 | 0.18 |
| tgfb | -0.00306 | 0.00186 | -1.65 | 0.0997 |
| tnfa | -0.0481 | 0.0379 | -1.27 | 0.205 |

**Additional file 1: Table S6. Correlation between BPF and MS-outcome.**

|  | estimate | Std.error | statistic | Pvalue |
| --- | --- | --- | --- | --- |
| GroupBenign | 0.0029 | 0.007 | 0.37 | 0.7 |

**Additional file 1: Table S7. Genes targeted by miR-320b and mIR-25-3p in the adherens junction pathway and hippo signaling pathway.**

|  | **Genes in adherens junction pathway** | | | **Genes in hippo signalling pathway** | | |  |
| --- | --- | --- | --- | --- | --- | --- | --- |
|  | **#** | **Gene Name** | **Gene Ensembl id** | **#** | **Gene Name** | **Gene Ensembl id** |  |
|  | **1.** | **ACTB** | [ENSG00000075624](http://www.ensembl.org/Multi/Search/Results?species=all;idx=gene;q=ENSG00000075624) | **1.** | **ACTB** | [ENSG00000075624](http://www.ensembl.org/Multi/Search/Results?species=all;idx=gene;q=ENSG00000075624) |  |
|  | **2.** | **TGFBR1** | [ENSG00000106799](http://www.ensembl.org/Multi/Search/Results?species=all;idx=gene;q=ENSG00000106799) | **2.** | **GSK3B** | [ENSG00000082701](http://www.ensembl.org/Multi/Search/Results?species=all;idx=gene;q=ENSG00000082701) |  |
|  | **3.** | **CTNND1** | [ENSG00000198561](http://www.ensembl.org/Multi/Search/Results?species=all;idx=gene;q=ENSG00000198561) | **3.** | **TGFBR1** | [ENSG00000106799](http://www.ensembl.org/Multi/Search/Results?species=all;idx=gene;q=ENSG00000106799) |  |
|  | **4.** | **SMAD2** | [ENSG00000175387](http://www.ensembl.org/Multi/Search/Results?species=all;idx=gene;q=ENSG00000175387) | **4.** | **YWHAH** | [ENSG00000128245](http://www.ensembl.org/Multi/Search/Results?species=all;idx=gene;q=ENSG00000128245) |  |
|  | **5.** | **ACTG1** | [ENSG00000184009](http://www.ensembl.org/Multi/Search/Results?species=all;idx=gene;q=ENSG00000184009) | **5.** | **SMAD2** | [ENSG00000175387](http://www.ensembl.org/Multi/Search/Results?species=all;idx=gene;q=ENSG00000175387) |  |
|  | **6.** | **SMAD3** | [ENSG00000166949](http://www.ensembl.org/Multi/Search/Results?species=all;idx=gene;q=ENSG00000166949) | **6.** | **PRKCI** | [ENSG00000163558](http://www.ensembl.org/Multi/Search/Results?species=all;idx=gene;q=ENSG00000163558) |  |
|  | **7.** | **TJP1** | [ENSG00000104067](http://www.ensembl.org/Multi/Search/Results?species=all;idx=gene;q=ENSG00000104067) | **7.** | **PPP2CA** | [ENSG00000113575](http://www.ensembl.org/Multi/Search/Results?species=all;idx=gene;q=ENSG00000113575) |  |
|  | **8.** | **FYN** | [ENSG00000010810](http://www.ensembl.org/Multi/Search/Results?species=all;idx=gene;q=ENSG00000010810) | **8.** | **NF2** | [ENSG00000186575](http://www.ensembl.org/Multi/Search/Results?species=all;idx=gene;q=ENSG00000186575) |  |
|  | **9.** | **NLK** | [ENSG00000087095](http://www.ensembl.org/Multi/Search/Results?species=all;idx=gene;q=ENSG00000087095) | **9.** | **YWHAG** | [ENSG00000170027](http://www.ensembl.org/Multi/Search/Results?species=all;idx=gene;q=ENSG00000170027) |  |
|  | **10.** | **CDH1** | [ENSG00000039068](http://www.ensembl.org/Multi/Search/Results?species=all;idx=gene;q=ENSG00000039068) | **10.** | **CCND2** | [ENSG00000118971](http://www.ensembl.org/Multi/Search/Results?species=all;idx=gene;q=ENSG00000118971) |  |
|  | **11.** | **CTNNB1** | [ENSG00000168036](http://www.ensembl.org/Multi/Search/Results?species=all;idx=gene;q=ENSG00000168036) | **11.** | **ACTG1** | [ENSG00000184009](http://www.ensembl.org/Multi/Search/Results?species=all;idx=gene;q=ENSG00000184009) |  |
|  | **12.** | **PTPRJ** | [ENSG00000149177](http://www.ensembl.org/Multi/Search/Results?species=all;idx=gene;q=ENSG00000149177) | **12.** | **GLI2** | [ENSG00000074047](http://www.ensembl.org/Multi/Search/Results?species=all;idx=gene;q=ENSG00000074047) |  |
|  | **13.** | **RAC1** | [ENSG00000136238](http://www.ensembl.org/Multi/Search/Results?species=all;idx=gene;q=ENSG00000136238) | **13.** | **FZD6** | [ENSG00000164930](http://www.ensembl.org/Multi/Search/Results?species=all;idx=gene;q=ENSG00000164930) |  |
|  | **14.** | **INSR** | [ENSG00000171105](http://www.ensembl.org/Multi/Search/Results?species=all;idx=gene;q=ENSG00000171105) | **14.** | **SMAD3** | [ENSG00000166949](http://www.ensembl.org/Multi/Search/Results?species=all;idx=gene;q=ENSG00000166949) |  |
|  | **15.** | **SSX2IP** | [ENSG00000117155](http://www.ensembl.org/Multi/Search/Results?species=all;idx=gene;q=ENSG00000117155) | **15.** | **WWTR1** | [ENSG00000018408](http://www.ensembl.org/Multi/Search/Results?species=all;idx=gene;q=ENSG00000018408) |  |
|  | **16.** | **EP300** | [ENSG00000100393](http://www.ensembl.org/Multi/Search/Results?species=all;idx=gene;q=ENSG00000100393) | **16.** | **CDH1** | [ENSG00000039068](http://www.ensembl.org/Multi/Search/Results?species=all;idx=gene;q=ENSG00000039068) |  |
|  | **17.** | **MAPK1** | [ENSG00000100030](http://www.ensembl.org/Multi/Search/Results?species=all;idx=gene;q=ENSG00000100030) | **17.** | **CCND1** | [ENSG00000110092](http://www.ensembl.org/Multi/Search/Results?species=all;idx=gene;q=ENSG00000110092) |  |
|  | **18.** | **CREBBP** | [ENSG00000005339](http://www.ensembl.org/Multi/Search/Results?species=all;idx=gene;q=ENSG00000005339) | **18.** | **CTNNB1** | [ENSG00000168036](http://www.ensembl.org/Multi/Search/Results?species=all;idx=gene;q=ENSG00000168036) |  |
|  |  |  |  | **19.** | **AXIN2** | [ENSG00000168646](http://www.ensembl.org/Multi/Search/Results?species=all;idx=gene;q=ENSG00000168646) |  |
|  |  |  |  | **20.** | **MYC** | [ENSG00000136997](http://www.ensembl.org/Multi/Search/Results?species=all;idx=gene;q=ENSG00000136997) |  |
|  |  |  |  | **21.** | **CSNK1E** | [ENSG00000213923](http://www.ensembl.org/Multi/Search/Results?species=all;idx=gene;q=ENSG00000213923) |  |
|  |  |  |  | **22.** | **WNT2** | [ENSG00000105989](http://www.ensembl.org/Multi/Search/Results?species=all;idx=gene;q=ENSG00000105989) |  |
|  |  |  |  | **23.** | **SMAD7** | [ENSG00000101665](http://www.ensembl.org/Multi/Search/Results?species=all;idx=gene;q=ENSG00000101665) |  |
|  |  |  |  | **24.** | **LATS1** | [ENSG00000131023](http://www.ensembl.org/Multi/Search/Results?species=all;idx=gene;q=ENSG00000131023) |  |
|  |  |  |  | **25.** | **LATS2** | [ENSG00000150457](http://www.ensembl.org/Multi/Search/Results?species=all;idx=gene;q=ENSG00000150457) |  |
|  |  |  |  | **26.** | **CTGF** | [ENSG00000118523](http://www.ensembl.org/Multi/Search/Results?species=all;idx=gene;q=ENSG00000118523) |  |
|  |  |  |  | **27.** | **PPP1CB** | [ENSG00000213639](http://www.ensembl.org/Multi/Search/Results?species=all;idx=gene;q=ENSG00000213639) |  |
